# Addressing the energy crisis: using microbes to make biofuels

**DOI:** 10.1111/1751-7915.14050

**Published:** 2022-03-17

**Authors:** Juan L. Ramos, Ben Pakuts, Patricia Godoy, Ana García‐Franco, Estrella Duque

**Affiliations:** ^1^ Estación Experimental del Zaidín CSIC Granada Spain; ^2^ University Health Network Toronto ON Canada

## Abstract

Much of the energy being used to power our lives comes from fossil fuels such as coal, natural gas and petroleum. These energy sources are non‐renewable, are being exhausted and also pollute the air, water and soil with toxic chemicals. Their mining, transportation, refining and use are associated with a large carbon footprint that contributes significantly to global warming. In addition, the geopolitical complexities surrounding the main fossil fuel producers create risks and uncertainties around the world. Replacing fossil fuels with clean, renewable forms of energy is paramount to creating a sustainable and healthy future, and for laying the foundations for global political stability and prosperity. Using biomass from plants, microbes can produce biofuels that are identical to or perform as well as fossil fuels. In addition of creating sustainable energy, advancing the biofuel industry will create new, high‐quality rural jobs whilst improving energy security.

## The need for renewable fuels

The industrial revolution at the end of the 19th century sparked the beginning of the modern era. Throughout the 20^th^ century and until now, huge economic and technological leaps have been made. Improvements in the production of goods and advances in medicine have contributed to increased life expectancy across the world. Increases in human population, combined with the development and expansion of terrestrial, maritime and air transportation, have led to a highly connected world and high energy demands.

With bans on the use of nuclear energy being enacted in some countries due to short‐ and long‐term safety concerns, and nations working to reduce reliance on fossil fuels (i.e. petroleum, coal and natural gas) due to their polluting effects, we need to find other ways to create energy. These alternative sources of energy can be sourced from sunlight (i.e. photovoltaic and thermosolar), wind, ocean tides and microbes. No single means of making energy is sufficient to cover the world’s energy demands; however, when developed and used concurrently, these approaches may be sufficient to meet current and future needs.

## Biofuels

The Kyoto Protocols and the Paris Climate Agreement call for the use of clean, green and renewable transportation fuels to replace gasoline, diesel and jet fuel. Biofuels are a promising alternative to fossil fuels and are produced from biological materials – most often from cereal grains, sugarcane or biomass derived from plants or wastes. They are considered renewable fuels because they originate from plant materials made via photosynthesis and sunlight‐powered CO_2_ fixation. Plants can be grown continually to provide a constant supply of raw materials, which is in contrast to the constantly diminishing supply of fossil fuels. Because plants use CO_2_ to grow, and CO_2_ is a greenhouse gas they sequester and reduce this greenhouse gas. However, their effect on net emissions must consider emissions caused by direct or indirect land use, the amount of carbon sequestered and the amount of greenhouse gases emitted through agriculture and biofuel production processes. In general, achieving carbon neutrality for biofuels requires high plant yields and low emissions (Hill *et al*., 2006; Fargioni *et al*., 2008; United Nations, 2016; Koçar and Civas, 2013).

A number of biofuel programmes have been implemented in the United States, Brazil and the European Union to reduce emissions and to reduce the importation of fossil fuels to enhance the security of national fuel supplies. Biofuels offer a number of social, economic, environmental and technical benefits, which include moderating oil prices, creating rural jobs, reducing global carbon emissions and decreasing soil erosion (Ramos *et al*., 2016; Valdivia *et al*., 2016). That said, controversies have arisen, such as the fear that the use of agricultural land for biofuel production will endanger food production (the ‘food versus fuel’ debate).

Despite the potential represented by biofuels, current estimates indicate that only about 1% of the energy used globally can be traced back to a biofuel source. Therefore, there exist great opportunities to increase the use of renewable fuels. More recent data suggest that, in certain sectors, biofuel use is gaining traction. Data from 2018 estimate that worldwide biofuel production reached 152 billion litres (40 billion gallons US) and provides about 3% of the world's fuel for road transport (Teter *et al*., 2019). Furthermore, to reduce dependency on petroleum, several international agencies and governments aim to use biofuels to supply 25% of their transportation energy by 2050.

## Crops for biofuels

A number of crops are grown specifically for biofuel production and are known as energy crops. They vary according to geography: for example, corn, soybeans, willows and switchgrass are common energy crops in the United States; rapeseed, wheat, sugar beet and willows are preferentially grown in northern Europe; sugarcane is grown in Brazil; palm oil and *Miscanthus giganteus* (giant silver grass) are grown in Southeast Asia; and sorghum and cassava are grown in China. Worldwide, corn grain and sugarcane are the most common biofuel crops, while Miscanthus is believed to be the most efficient biofuel crop. In addition to plant material, algae and the organic fraction of municipal solid waste are also considered feedstocks for biofuel production.

## First‐generation (1G) and second‐generation (2G) biofuels

Depending on the source of the feedstock, biofuels are referred to as either first, second, third or fourth generation. First‐generation biofuels are conventional biofuels made from food crops grown on arable land (Mohr and Raman, 2013). The sugar, starch or vegetable oil obtained from the crops is converted into biodiesel or ethanol. This can occur via transesterification (biodiesel), or via fermentation mediated by yeast or bacteria. The impetus behind advancing first‐generation biofuels to market was driven by the need to reduce greenhouse gas emissions, stabilize oil prices and increase energy security. However, first‐generation biofuels became marred in controversy because, as competitors for arable land, they were soon perceived as a threat to agricultural food production and food security. To overcome these challenges, the industry has focussed on the development of biofuels using alternative feedstocks and new innovative technologies, which have given rise to second‐generation biofuels.

Second‐generation biofuels are made from lignocellulosic biomass or woody crops, agricultural waste and the organic fraction of municipal solid waste (MSW). Therefore, the feedstocks used to generate second‐generation biofuels are either by‐products of food crops, are grown on land that is not appropriate for food crops or derived from organic wastes.

Selecting the land on which to grow a feedstock is a critical determinant of the sustainability of the biofuel, and a key consideration is the minimization of the competition between biofuel production and food production.

The use of municipal and household waste for biofuel production is an emerging approach – one that makes use of lignocellulose that is currently a largely unused resource. The use of this biomass as an energy source has the potential to improve waste management, fuel security and help address climate change (Valdivia *et al*., 2020).

Third‐generation biofuels are derived from algae, while fourth‐generation biofuels include electro‐fuels and photobiological solar fuels (Hannon *et al*., 2010; Singh and Yakhmi, 2014). Third‐ and fourth‐generation technologies are under development and not yet market‐ready at the industrial scale.

## Types of biofuels

All biofuels can be made as liquids (alcohols and biodiesel) or gas (biogas).

Currently, bioethanol is the most relevant biologically produced commodity. Almost all of the ethanol used in the world for pharma, solvent industries and fuels is produced through biological fermentation. Bioethanol is a 1G fuel that is commonly produced worldwide, particularly in Brazil and the United States. Alcohol fuels are produced through the fermentation of sugars derived mainly from corn grain and sugarcane, as well as from sugar beet, wheat grain (or other cereal grains), molasses and various other plants, including fruit and fruit waste.

In the case of grain, the first step of biofuel production is the hydrolysis of starch using amylases. This process produces simple sugars – mainly glucose – which are then fermented to ethanol using microorganisms such as yeasts or bacteria (e.g. *Zymomonas*). In the United States, ethanol production rates are in the range of 14–15 billion gallons per year at corn dry mills. These mills produce not only ethanol but also corn oil and dry distillers’ grains, which are used as animal feed. The CO_2_ produced during fermentation is harvested and used for carbonated drinks or for medical uses. In Brazil, about 5 billion gallons of ethanol are produced annually and the leftover waste (i.e. bagasse) is often burnt in the mills to generate extra energy. This first‐generation ethanol technology is quite mature, and industrial ethanol plants are usually profitable.

Ethanol can be used as a fuel for vehicles in its pure form (E100), but it is usually used as a gasoline additive to increase octane rate and decrease vehicle emissions. Modern car petrol engines can run on blends of up to 10% (v/v) bioethanol with gasoline (E10); however, it should be noted that ethanol has a smaller energy density than gasoline. For this reason, it takes more fuel (volume and mass) to produce the same amount of work (Elfasakhany, 2015).

Despite the success of 1G bioethanol, it was calculated that if all the corn in the United States were used to produce biofuels, it would only satisfy 12% of the demand for gasoline (Hill *et al*., 2006). Thus, the industry has begun looking at using agricultural waste leftover after harvest and cellulosic material present in MSW as source materials for bioethanol production.

The production of 2G bioethanol is well‐developed and requires three major steps: (i) the physicochemical pre‐treatment of the lignocellulosic biomass to make polysaccharides (cellulose and hemicellulose) accessible; (ii) the enzymatic breakdown of cellulose and hemicellulose into constituent sugars; and (iii) the fermentation of sugars using specialized yeasts or bacteria.

At present, the main hurdles facing 2G ethanol seem to arise from mechanical issues in the handling of materials and the efficient operation of the pre‐treatment units. Another relevant hurdle is the price of the enzymatic cocktail – at present, this costs more than amylases for 1G by an order of magnitude. The main source of these cocktails are enzymes secreted by fungi, which use them to metabolize lignocellulosic residues present in plant material, such as leaf litter and dead wood. These enzymatic cocktails enable the release >80% of the monosaccharide sugars that are present in celluloses and hemicelluloses (Alvarez *et al*., 2016). Most of the sugar used to produce ethanol in 1G processes is glucose. While glucose is also the predominant sugar in 2G processes (approximately 75% of total sugars), significant amounts of other sugars are also involved, including xylose (23%) and arabinose. Fermentation of sugars released from corn stover, bagasse and other agricultural residues requires the use of specialized yeasts that can simultaneously ferment glucose and xylose. The yeasts used in 2G fermentation are genetically modified to convert more than 96% of glucose and more than 90% of xylose to ethanol with overall fermentation yields >90% of the theoretical maximum – an achievement that demonstrates how far this technology has progressed.

Another source of cellulosic material is the organic fraction recovered from MSW. The technologies used in these processes are very similar to those used for the production of ethanol from corn stover or bagasse, with the added requirement for a series of early steps to separate the organic fraction from other materials present in the waste biomass.

Solid waste management contributes around 5% to global greenhouse gas emissions, and Turner *et al*. (2015) stressed the importance of ensuring that MSW is sustainably sourced. If MSW is properly sourced, its use could reduce greenhouse gas emissions by 65%, even when considering all possible indirect emissions. In the United States, the organic fraction comprises about 61% of total weight of MSW according to the EPA. If the 164 million tons that are currently diverted to landfills were converted to bioethanol, about 7.5 billion gallons of ethanol could be produced – the equivalent of about 250 million barrels of petrol. Furthermore, it has been estimated that biofuels from MSWs and agricultural residues could replace 16% of fuel used by the United States transportation sector by 2030. By looking towards 2050, there is great potential for the production of biofuels from non‐edible plant materials and MSW residues.

After ethanol, butanol is the most promising biofuel. Biobutanol is often cited as a potential replacement for gasoline because it can be used directly in internal combustion engines. In the past, butanol was produced through what is known as ABE fermentation, an anaerobic process efficiently carried out by a number of strains of the genus *Clostridium* that yield a mixture of acetone, butanol and ethanol at a ratio of 1:6:1 (Qureshi and Blaschek, 2008; Green, 2011). Although ABE fermentation has since been replaced by the chemical production of butanol from petrol, a number of research efforts have been initiated to increase the proportion of butanol that can be produced through fermentation – efforts that have led to processes capable of yielding an ABE ratio of 1:8:1 (Jang *et al*., 2012). These gains have been achieved through the development of *Clostridium* strains with a genetically modified metabolic pathway, as well as through selecting strains that are able to withstand higher butanol concentrations. With further research, technical advancements and industry investments, biobutanol has the potential to become more profitable than ethanol. Moreover, a number of clostridia strains are able to degrade lignocellulose material (i.e. from agricultural waste and forestry residues), suggesting that a second‐generation biobutanol industry is feasible.

Those who support a shift to butanol production point to three key benefits: (i) butanol has a higher fuel density than ethanol and is less corrosive; (ii) it can be added to gasoline at a higher blend ratio, the so‐called ‘BUT16’; and (iii) it is highly compatible with existing petroleum distribution systems, including fuel pumps.

## Biodiesel

Biodiesel, another transportation fuel, can be produced from leftover food products. Biodiesel is the most common biofuel in Europe and is generated through a process that involves transesterification of vegetable oils or animal fats. It can be used as a fuel for vehicles in its pure form (B100), and because biodiesel is an oxygenated fuel and has a higher hydrogen and oxygen content than standard diesel. Because of this, its combustion leads to lower particulate and carbon monoxide emissions. However, using pure biodiesel may increase emissions of the greenhouse gas nitrous oxide. Biodiesel is mainly produced from vegetable oils, and microbes play only a minor role in the process. Nonetheless, it should be mentioned that several fungi accumulate large amounts of fatty acids that are easily converted into alkanes. One of these oleaginous fungi is a strain of *Aspergillus* that can produce 20% more fatty acids than normal fungi. Some strains of this fungus are able to make fatty acids directly from free sugars and cellulosic materials (Subhash and Mohan, 2011).

## Biogas

Biodegradable outputs from industry, agriculture, forestry and households can be used for biofuel production through anaerobic digestion to produce biogas. Anaerobic biogas production is catalysed by methanogens, which digest material inside a closed system, known as anaerobic digester, bio‐digester or a bioreactor (Richards *et al*., 1994). Biogas is primarily methane (CH_4_) and carbon dioxide (CO_2_), and methane can be combusted or oxidized with oxygen, releasing energy. Advanced waste treatment technologies can produce biogas comprising 55%–75% methane. Reactors with free liquids can produce gas comprising 80%–90% methane using *in situ* gas purification techniques. Biogas is a renewable energy source because its production and use cycle are continuous, and the process generates no net carbon dioxide. Furthermore, the solid by‐product, which is known as digestate, can be used as a fertilizer. Biogas can also be compressed after the removal of carbon dioxide and used to power motor vehicles, and it can be cleaned and upgraded to meet natural gas standards.

### Relevance for sustainable development goals and grand challenges

Biofuels represent a set of renewable fuels that could replace fossil fuels. The combustion of biofuels is cleaner than fossil fuels and also serves to reduce the emission of toxic chemicals. Biofuels also promote the use of land and marginal lands to grow energy crops, which promotes the creation of high‐quality and stable rural jobs. Bioethanol and biodiesel are currently players in the market and have the capacity to replace gasoline and diesel in combustion motors.

The value of biofuels goes beyond their use as transportation fuels, and attention should be given to the economic and environmental benefits of the co‐products of biofuels. Presently, the long‐term success of 2G biofuels requires financial incentives and supportive regulations, which are instrumental for driving the commercial production and adoption of advanced biofuels.

The field of biofuel research is an exceptionally dynamic and exciting arena that has the potential to transform how we produce energy. It holds the key to creating a more sustainable and circular economy and relies on starting materials that are currently considered waste. There is an enormous impetus for the development of affordable genomics technologies, and these are going to be critical for next‐generation fuels. The revolution in synthetic biology is enabling the development of novel biofuels capable of replacing kerosene. These new fuels will help ameliorate CO_2_ emissions from a variety of human activities, including transportation and air flight. Biofuels are highly aligned with UN SDG targets because they have the potential to reduce greenhouse gas emissions, reduce pollution, promote energy security and create stable and high‐quality rural jobs.

As part of the UN’s global development agenda, and as outlined in the latest summit outcome document, SDGs serve as a cornerstone initiative until 2030. While SDGs are not legally binding treaties, their realization is driven by moral and political commitments. One of these agreements compels UN countries to move towards the responsible use of energies and the replacement of fossil fuels by green renewable sources such as biofuels.

## Conflict of interest

None declared.
